# Role and prognostic significance of the epithelial-mesenchymal transition factor ZEB2 in ovarian cancer

**DOI:** 10.18632/oncotarget.3943

**Published:** 2015-05-15

**Authors:** Silvia Prislei, Enrica Martinelli, Gian Franco Zannoni, Marco Petrillo, Flavia Filippetti, Marisa Mariani, Simona Mozzetti, Giuseppina Raspaglio, Giovanni Scambia, Cristiano Ferlini

**Affiliations:** ^1^ Department of Obstetrics and Gynecology, Catholic University of the Sacred Heart, Rome, Italy; ^2^ Department of Pathology, Catholic University of the Sacred Heart, Rome, Italy; ^3^ Reproductive Tumor Biology Research, Biomedical Lab, Department of Obstetrics and Gynecology, Danbury Hospital, Danbury, CT, USA

**Keywords:** ZEB2, EMT, HuR, ovarian cancer

## Abstract

ZEB2 is a key factor in epithelial-mesenchymal transition (EMT), a program controlling cell migration in embryonic development and adult tissue homeostasis. We demonstrated a role of ZEB2 in migration and anchorage-independent cell growth in ovarian cancer, as shown by ZEB2 silencing. We found that the RNA-binding protein HuR bound the 3′UTR of ZEB2 mRNA, acting as a positive regulator of ZEB2 protein expression. In Hey ovarian cell line, HuR silencing decreased ZEB2 and ZEB1 nuclear expression and impaired migration. In hypoglycemic conditions ZEB2 expression decreased, along with ZEB1, vimentin and cytoplasmic HuR, and a reduced cellular migration ability was observed. Analysis of ZEB2 and HuR expression in ovarian cancers revealed that nuclear ZEB2 is localized in tumor leading edge and co-localizes with cytoplasmic HuR. In a series of 143 ovarian cancer patients high expression of ZEB2 mRNA significantly correlated with a poor prognosis in term of both overall survival and progression- free survival. Moreover, at immunohistochemical evaluation, we found that prognostic significance of ZEB2 protein relies on its nuclear expression and co-localization with cytoplasmic HuR. In conclusion our findings indicated that nuclear ZEB2 may enhance progression of EMT transition and acquisition of an aggressive phenotype in ovarian cancer.

## INTRODUCTION

Ovarian cancer is the most lethal gynecologic malignancy, primarily for the advanced stage at diagnosis and the recurrence of chemotherapy-resistant tumors [1–2]. Unlike cancer in other organs site, ovarian carcinoma can spread by direct extension to adjacent organs, and exfoliated tumour cells can be transported throughout the peritoneal cavity by normal peritoneal fluid [3]. Hence, elucidating the mechanism involved in ovarian carcinoma invasion and progression is crucial for the development of targeted therapy.

The migration and invasiveness of the epithelial tumor cells depends on the activation of a reversible development process called epithelial-to-mesenchymal transition (EMT) [4]. During carcinogenesis, normal epithelial cells lose their cell polarity and cell–cell adhesion, but gain migration and invasiveness capacity during the process of malignant transformation thus invading surrounding tissues and distant sites. Once the cells reach their new niche, they can activate the reverse program – mesenchymal-to-epithelial transition (MET) – to form metastasis, as described in ovarian cancer [5]. In different systems it was clarified that these dramatic changes in cell behavior are triggered in response to extracellular signals, like TGF-β, or protein misregulation. Several layers and networks of regulation can be altered, including the transcriptional and translational machinery, expression of non–coding RNA, alternative splicing and protein stability [6–7]. Nevertheless, the strength of EMT is primarily dependent on the potency of EMT-inducing transcription factors (EMT-TFs) represented by several protein families, such as SNAIL, ZEB, or TWIST. Among them, the transcriptional repressor ZEB2 was deeply studied proving to be not only an EMT activator but also a key factor in promoting the initiation and development of different tumors [8–13]. ZEB2 gene expression was described to be regulated at post-transcriptional levels by the activity of several miRNAs, five of them corresponding to the miR-200 family [14–16, 7]. Expression of the miR-200 family is strongly associated with epithelial differentiation, and a reciprocal feedback loop between the miR-200-family and and the ZEB family tightly controls both EMT and MET.

In this context, the ubiquitous RNA-binding protein HuR has been demonstrated to regulate several mRNAs encoding proteins implicated in carcinogenesis [17–18], through the association with AU-and U-rich elements (AREs) in the 3′UTR of target mRNAs. It has been proposed that HuR can exert a central tumorigenic activity by enabling multiple cancer phenotypes, as promotion of cell proliferation, enhancement of cell survival, elevation of local angiogenesis, evasion of immune recognition, invasion and metastasis. The function of HuR is controlled at multiple levels, being relevant not only the quantity and integrity of HuR protein, but also the cellular localization. In particular, the increased cytoplasmic accumulation of HuR observed in both patient tumors and cancer cells correlates with an increased stabilization of mRNAs encoding cancer-related proteins [17–18].

The aim of our study was to further analyze the role of ZEB2 in the development of ovarian cancer and to define its prognostic significance. We demonstrated here the impact of ZEB2 on migration and anchorage-independent cell growth in Hey cancer cells, and we esteblished the functional association between HuR and ZEB2. Moreover, the nuclear expression of ZEB2 was found to be of relevant prognostic significance in ovarian cancer.

## RESULTS

### Expression of ZEB2 in ovarian adenocarcinoma cell lines

**Figure 1:**
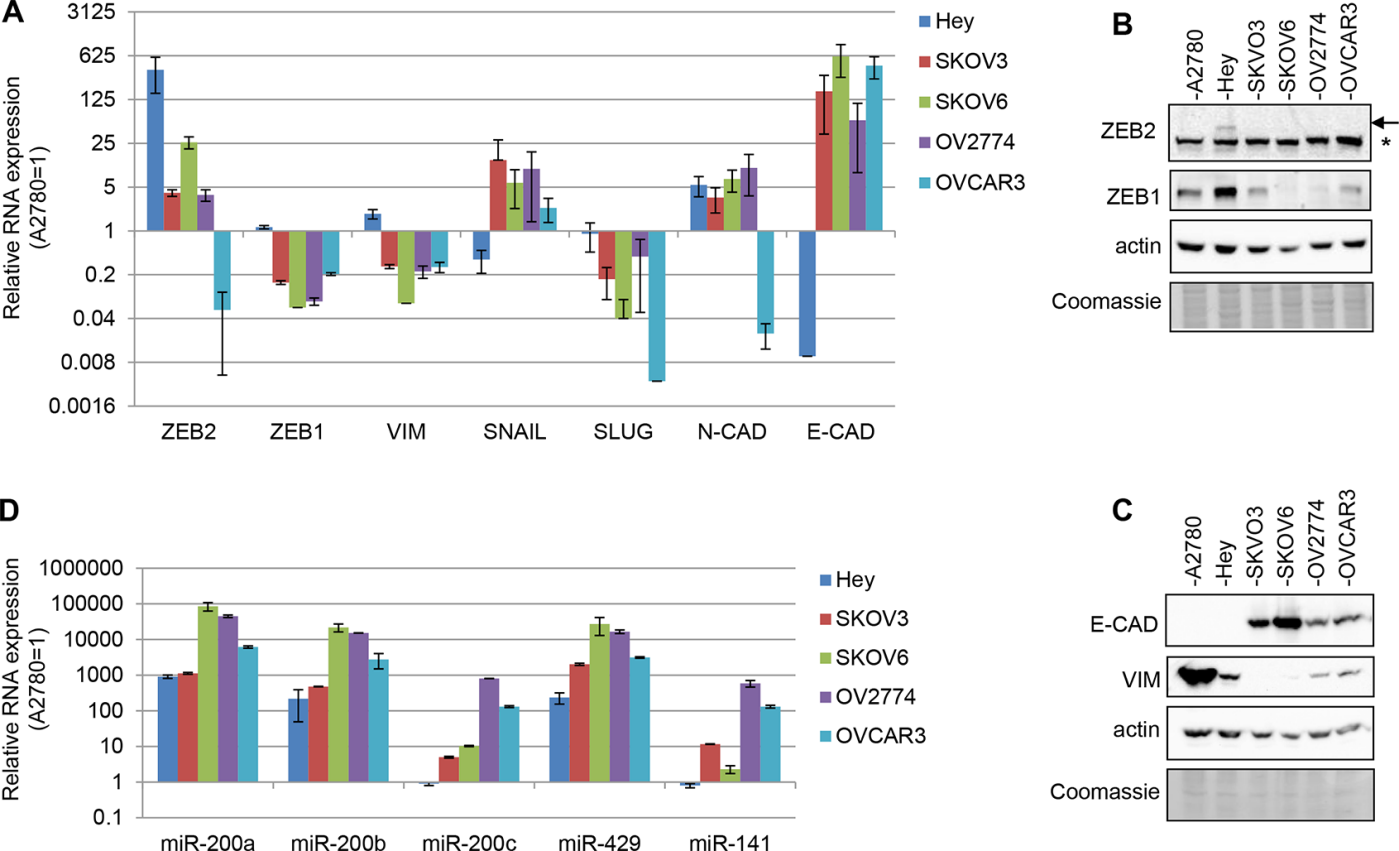
Expression of EMT markers in ovarian adenocarcinoma cell lines **A.** Q-PCR analysis of ZEB2, ZEB1, vimentin, SNAIL, SLUG, N-cadherin and E-cadherin mRNAs expression in A2780, SKOV3, SKOV6, OV2774 and OVCAR3 cell lines. **B.** and **C.** Representative Western Blots for EMT markers, respectively on nuclear lysates (B) and total lysates (C) in the cell lines indicated. The arrow indicated ZEB2 protein band and the asterisk indicated an unspecific band. Actin probing served as loading control. The Coomassie staining was used as additional loading control. **D.** Q-PCR analysis of miR-200a, miR-200b, miR-200c, miR-429 and miR-141 expression in cell lines as in (A) In bar charts (A) and (B) expression was normalized on the levels measured in A2780 cells (=1); bars and error bars refer to mean and SD of two experiments performed in triplicate.

In order to expanding the study of ZEB2 role in ovarian cancer, the expression of ZEB2 gene was firstly assessed in a panel of ovarian adenocarcinoma cell lines (A2780, Hey, SKOV3, SKOV6, OV2774, OVCAR3), along with additional EMT markers. ZEB2 mRNA was highly expressed in Hey cells, together with the mesenchymal genes ZEB1, SLUG and vimentin, while the mRNA of the epithelial marker E-cadherin was almost undetectable in Hey cell line (Figure 1A). Figure 1B showed that ZEB2 protein is detectable in our panel only in Hey cells; the arrow pointed the 150kD ZEB2 protein, while the asterisk indicated an unspecific band, present even in OVCAR3 sample, where ZEB2 mRNA expression is almost undetectable. The protein expression of vimentin and ZEB1, and the absence of E-cadherin protein was confirmed in Hey cells (Figure 1B and 1C).

The miR-200 family plays an important role in down-regulation of ZEB1/ZEB2 expression, being in turn repressed by these factors, in a well described inihibitory feedforward loop [7]. Therefore with the aim of analyzing the correlation of expression among these genes we analyzed the expression of miR-200a, miR-200b, miR-200c, miR-429, miR-141 in our panel of cell lines (Figure 1D). In Hey cells, where ZEB2 expression is not repressed (Figure 1A and 1B), particularly miR-200c and miR-141 appeared down-regulated, suggesting a correlation among the expression of these genes. Moreover, the results indicated that the expression of the miRNAs of the family varied considerably within all the cell lines analyzed, supporting the notion of specific roles played by the different family members and the presence of a spectrum of EMT phenotypes.

### ZEB2 and ZEB1 are involved in the migration and anchorage-independent cell growth in Hey cell line

Since it is well recognized that EMT is involved in cell migratory capacity in different carcinomas [4], we explored the role of ZEB2 in this specific context.

**Figure 2:**
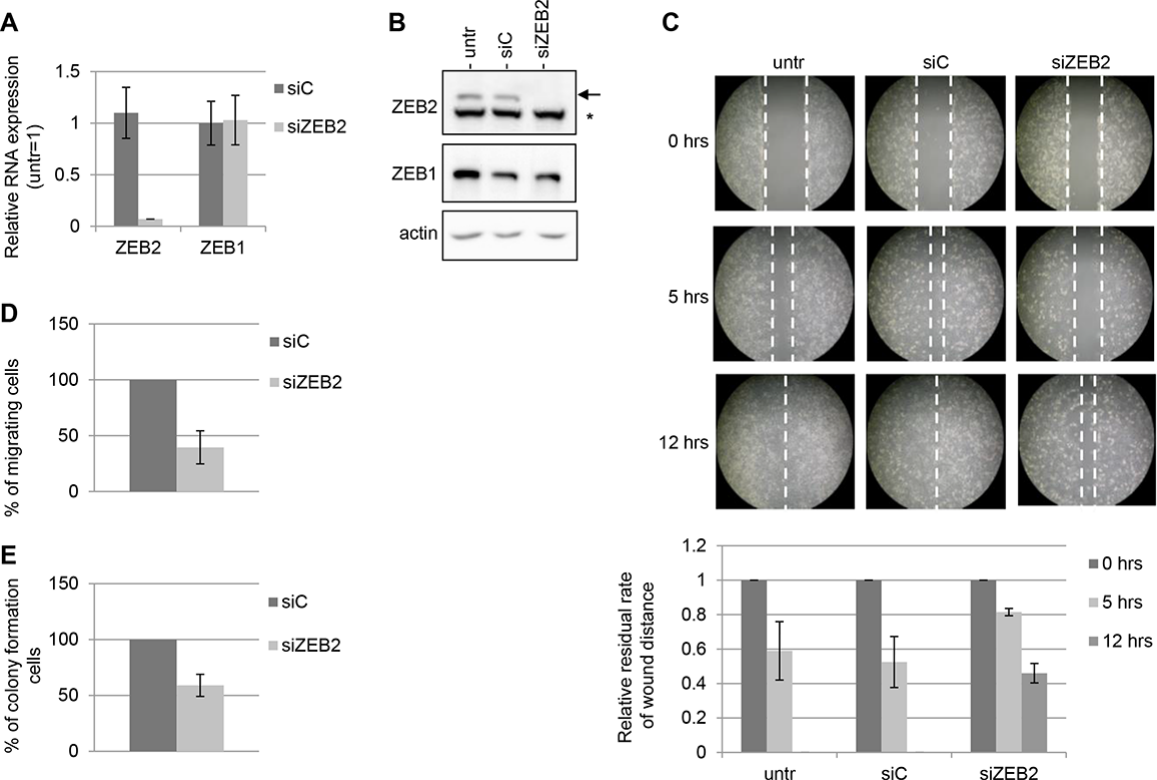
ZEB2 knockdown impairs migration and anchorage-independent cell growth in Hey cells The experiments were performed on Hey cells untrasfected, trasfected with siZEB2 oligos or with siC oligos and incubated for 48 hours. **A.** Q-PCR analysis of ZEB2 and ZEB1 mRNAs expression, values are expressed relative to the levels measured in untrasfected cells (=1). Bars and error bars refer to mean and SD of two experiments performed in triplicate. **B.** Representative Western Blots analysis of ZEB2 and ZEB1 protein expression on nuclear extracts. The arrow indicated ZEB2 protein band and the asterisk indicated an unspecific band. Actin probing served as loading control. **C.** Representative wound healing assay; the extent of closure of the wound, representing cell migration, was monitored under phase-contrast microscopy and images were captured at 0, 5 and 12 hours. Five fields in each 100 mm-plate were monitored and the experiments were performed twice. Wound closure was measured and represented in graphical format, with bars and error bars referring to mean and SD. The residual rate of wound distance is relative to time zero. **D.** Transwell migration assays. The values are expressed as percentage of migrating cells relative to siC-transfected cells. Bars and error bars refer to mean and SD of two experiments performed in duplicate. **E.** Anchorage-independent cell growth assays. The values are expressed as percentage of colonies relative to siC-transfected cells. Bars and error bars refer to mean and SD of two experiments performed in duplicate.

ZEB2 expression was knocked down in Hey cell line, where the silencing with siZEB2 oligos was achieved both at mRNA and protein level, respect to the control with siC oligos (Figure 2A and 2B). To confirm that the silencing was specific for ZEB2 gene, in the same experiments we analyzed the expression of ZEB1, which is structurally and functionally related with ZEB2, whose levels resulted unaltered in transfected cells (Figure 2A and 2B).

To investigate the effect of ZEB2 silencing on cell migration, wound healing assay were performed and the results showed that Hey cells transfected with siZEB2 oligos displayed reduced migration abilities compared with the controls (Figure 2C). The decrease of migration is evident after 5 and 12 hrs of incubation. Furthermore, a transwell migration assay showed a similar reduction of migratory capacity (Figure 2D). After 5 hrs of incubation a decrease of 60% was observed in the number of migrated cells transfected with siZEB2 oligos.

To further explore the functional consequences of ZEB2 knock down, we evaluated the anchorage-independent cell growth capacity in the same silenced cells. Silencing of ZEB2 clearly decreased the number of colonies observed in soft agar, as compared with siC transfected cells (Figure 2E).

Our findings indicated that ZEB2 contributed to the migratory and anchorage-independent cell growth abilities independently from ZEB1, whose expression was unaltered in ZEB2-silenced cells. To search for specific functions of ZEB2 and ZEB1, the knockdown of ZEB1 in Hey cell line was performed and the effects were investigated. As shown in the Supplementary Figure 1, ZEB2 expression levels did not change in ZEB1-silenced cells, while the migration and anchorage-independent cell growth clearly decreased.

Overall our results demonstrated that both ZEB2 and ZEB1 contributed to the migratory and anchorage-independent cell growth abilities in Hey ovarian cancer cell line.

### The RNA-binding protein HuR interacts with ZEB2 mRNA

**Figure 3:**
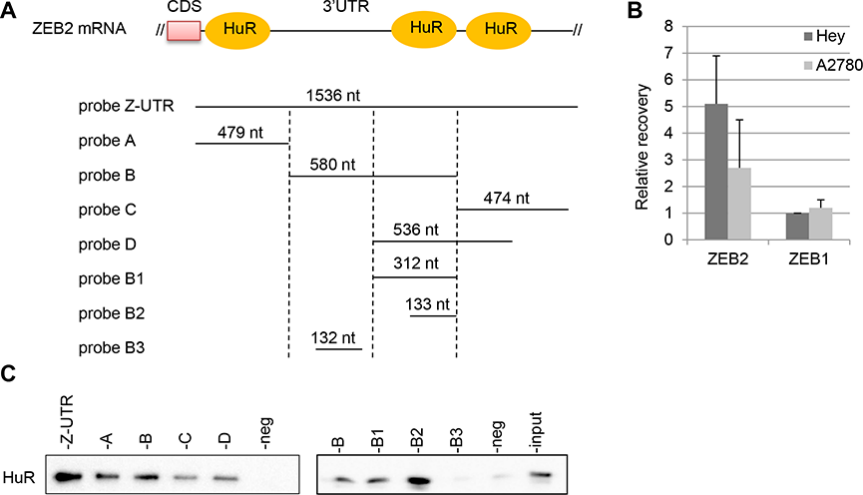
HuR associates with ZEB2 mRNA **A.** Schematic representation of the beginning sequence of ZEB2 3′UTR and of the RNA probes utilized for the pull-down experiments. The predicted sites of HuR binding were shown and the length of the the probes were indicated. **B.** RIP assay performed on Hey and A2780 cell lines with anti-HuR antibody. The recovery of ZEB2 and ZEB1 mRNAs from the input RNAs were quantified, normalizing the values for the unspecific IgG RIP and for the control HPRT mRNA. Bars and error bars refer to mean and SD of two experiments performed in triplicate. **C.** Pulldown assays, with biotinylated RNAs spanning different segments of the 3′UTR, as depicted in (A). The association of HuR with these probes was detected by Western Blot analysis. The negative control and the input extract were indicated.

Since ZEB2 gene is modulated at post-transcriptional level by miRNAs activity [7] and it is well known the functional interplay between miRNAs and RNA-binding proteins [19], we set out to investigate if ZEB2 can be modulated by RNA-binding proteins. In particular, HuR regulates the expression of several genes implicated in establishing cancer traits [17–18] and *in silico* predictions from RBPDB database showed several possible interaction sites through the 3′UTR region of ZEB2 mRNA, mostly at the beginning of the 3′UTR (Figure 3A). To study whether HuR is involved in modulation of ZEB2 expression, we examined the interaction of HuR with ZEB2 mRNA.

First, we performed ribonucleoprotein-immunoprecipitation (RIP) assay with anti-HuR antibody, under conditions that preserved the integrity of the RNA-protein complexes, in Hey and A2780 cell lines. The recovered RNAs from the immunoprecipitated material was subjected to reverse transcription and the association of HuR with ZEB2 mRNA was monitored through real-time quantitative PCR. As shown in Figure 3B, in Hey cells ZEB2 mRNA was enriched 5-fold in HuR RIP samples. Compared with this value, ZEB2 mRNA recovery was poorer in A2780 cells, expressing low levels of ZEB2 mRNA (see Figure 1A). The association of HuR with ZEB1 mRNA was also evaluated, as the 3′UTR of ZEB1 mRNA showed several predicted HuR binding sites according to RBPDB database (data not shown), but the recovery of ZEB1 mRNA was low, as for the control unrelated HPRT mRNA (Figure 3B).

Second, we studied if endogenous HuR bound to ectopic ZEB2 mRNAs by using the biotin pulldown assay. Biotinylated RNA probes spanning different regions of the 3′UTR sequence were synthesized (Figure 3A), incubated with cytoplasmic protein extracts of Hey cells and pulled down with streptavidin-conjugated agarose beads. Western blot analysis showed that probes Z-UTR, A, B, C, D, B1 and B2 were able to associate with HuR protein, although with different affinity (Figure 3C). The probe B3 did not include predicted binding site for HuR and was not bound by HuR, as well as the negative control. On the contrary the short probe B2, spanning positions 900 to 1033 of the 3′UTR, was still bound by HuR. Overall, our findings indicated that HuR associated with ZEB2 mRNA in Hey cell line.

### HuR regulates ZEB2 expression and affects cellular migration

To test the functional consequences of the interaction between HuR and ZEB2 mRNA, we studied if reduction of HuR abundance in Hey cells affected ZEB2 expression.

**Figure 4:**
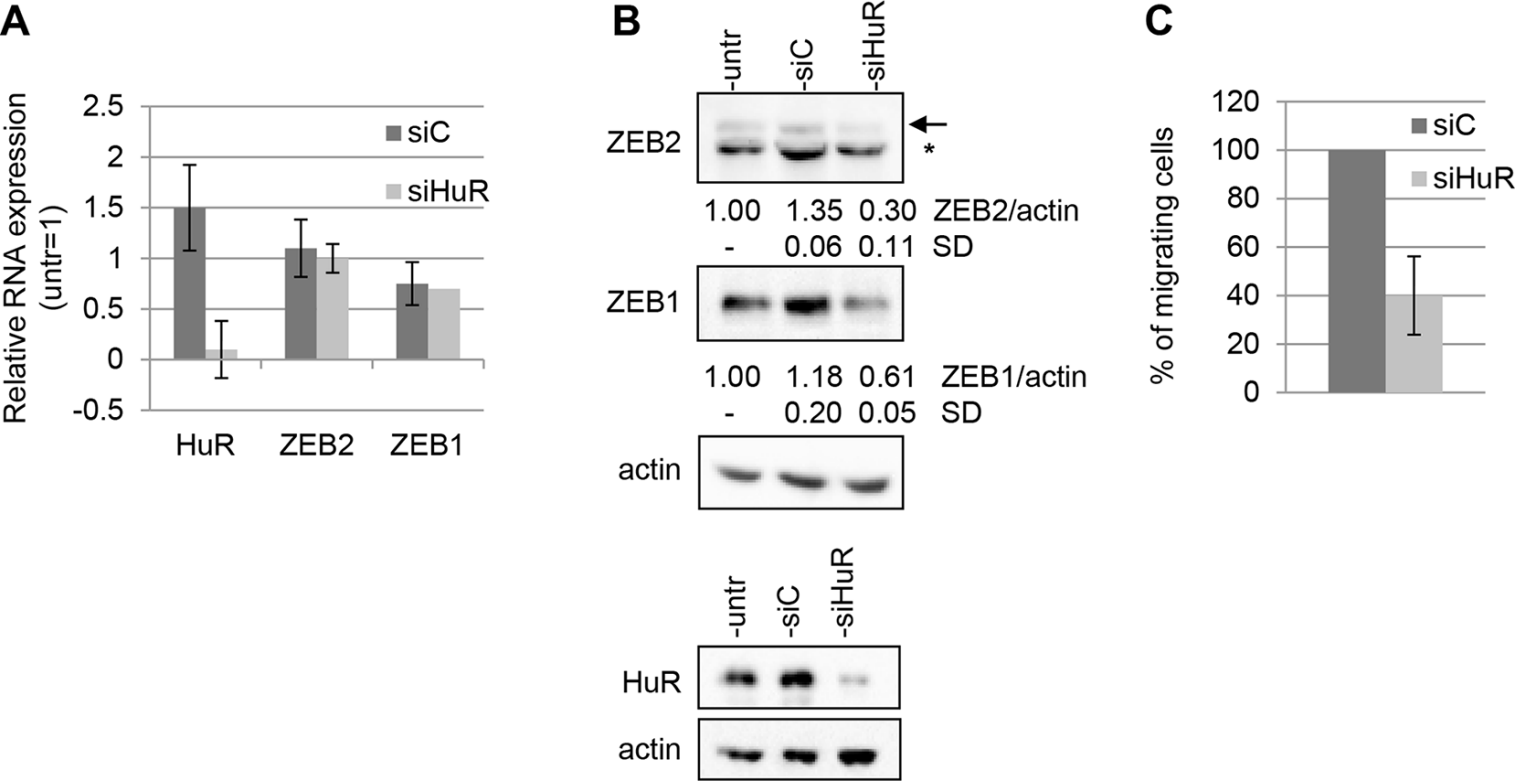
HuR modulates ZEB2 expression and affects cellular migration The experiments were performed on Hey cells untrasfected, trasfected with siHuR oligos or with siC oligos and incubated for 48 hours. **A.** Q-PCR analysis of HuR, ZEB2 and ZEB1 mRNAs expression, values are expressed relative to the levels measured in untrasfected cells (=1). Bars and error bars refer to mean and SD of three experiments performed in triplicate. **B.** Representative Western Blots analysis of ZEB2 and ZEB1 protein expression on nuclear extracts and of HuR protein expression on total extracts. The arrow indicated ZEB2 protein band and the asterisk indicated an unspecific band. Actin probing served as loading control. The values from densitometric analysis of the bands were normalized for actin and expressed relative to untrasfected cells. Standard deviations (SD) are also indicated. **C.** Transwell migration assays. The values are expressed as percentage of migrating cells relative to siC-transfected cells. Bars and error bars refer to mean and SD of two experiments performed in duplicate.

As shown, HuR silencing with specific siHuR oligos was achieved in Hey cells (Figure 4A and 4B). The analysis of ZEB2 expression in these cells indicated that HuR decrease did not significantly reduce ZEB2 mRNA abundance (Figure 4A), nevertheless determining an average of 4-fold reduction of ZEB2 protein expression (Figure 4B). These findings revealed that HuR positively regulates ZEB2 expression, either increasing ZEB2 mRNA stability or promoting the translation of the protein.

Moreover, we investigated whether HuR may play a role in the modulation of the related ZEB1 gene expression, although a direct association of HuR and ZEB1 mRNA failed to be detected by the RIP assay (see Figure 3B). The results showed that HuR silencing decreased ZEB1 protein expression without altering ZEB1 mRNA levels (Figure 4A and 4B), although the mechanism of ZEB1 expression modulation by HuR remains to be elucidated. Since we found that the decrease of ZEB2 expression resulted in an impaired migration ability of Hey cells (Figure 2D), we explored if the same effect could be observed reducing HuR levels. The results confirmed this hypothesis, demonstrating that Hey cell line transfected with siHuR oligos displayed a sharp decrease of cellular migration compared to the siC control (Figure 4C).

### Hypoglicemia reduces ZEB2 and ZEB1 abundance and cellular migration

**Figure 5:**
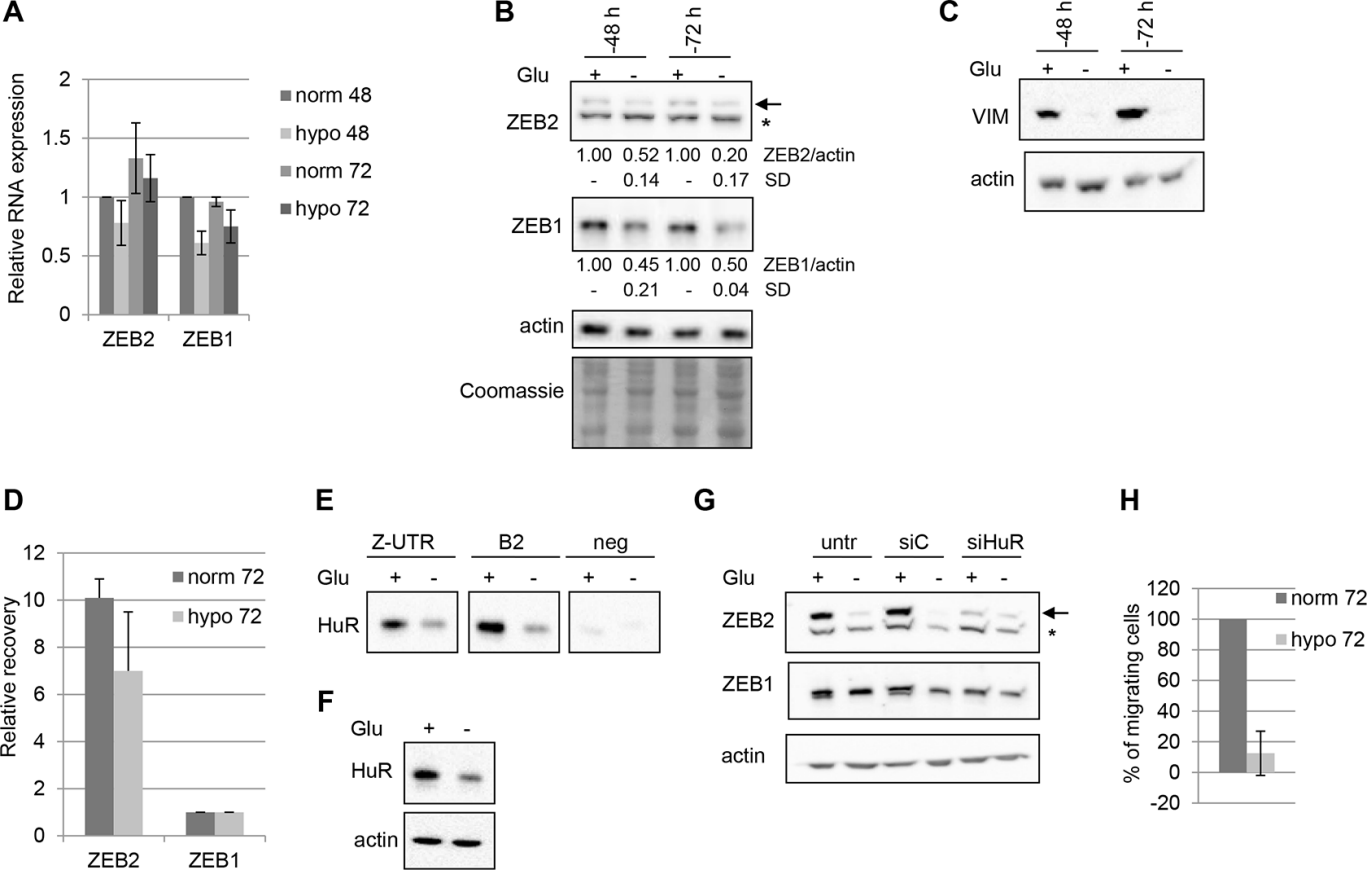
Hypoglycemia decreases ZEB2 levels and cellular migration The experiments were performed on Hey cells incubated in normoglycemic or hypoglycemic conditions for 48 or 72 hours as indicated. **A.** Q-PCR analysis of ZEB2 and ZEB1 mRNAs expression. Values are expressed relative to the levels measured in normoglycemia at 48 hours (=1). Bars and error bars refer to mean and SD of three experiments performed in triplicate. **B.** Representative Western Blots analysis of ZEB2 and ZEB1 protein expression on nuclear extracts and of HuR protein expression on total extracts. The arrow indicated ZEB2 protein band and the asterisk indicated an unspecific band. Actin probing served as loading control. The values from densitometric analysis of the bands were normalized for actin and expressed relative to to the levels measured in normoglycemia at 48 hours. Standard deviations (SD) are also indicated. The Coomassie staining was used as additional loading control. **C.** Representative Western Blots analysis of vimentin expression on total extracts. **D.** RIP assay performed on Hey cell line incubated in normoglycemia or hypoglycemia for 72 hours, utilizing with anti-HuR antibody. The recovery of ZEB2 and ZEB1 mRNAs from the input RNAs were quantified, normalizing the values for the unspecific IgG RIP and for the control HPRT mRNA. Bars and error bars refer to mean and SD of two experiments performed in triplicate. **E.** Pulldown assays, with ZUTR, B2 and negative control biotinylated RNAs, as depicted in Figure 3A. The association of HuR with these probes was detected by Western Blot analysis. **F.** Representative Western Blots analysis of HuR expression on cytoplasmic extracts utilized for the pull-down assays. **G.** Representative Western Blots analysis of ZEB2 and ZEB1 protein expression on nuclear extracts of Hey cells untrasfected, trasfected with siHuR oligos or with siC oligos, incubated for 6 hours, then treated in ipoglycemic conditions for 72 hours, as indicated. Efficient silencing of HuR were controlled by Q-PCR (data not shown). **H.** Transwell migration assays. The values are expressed as percentage of migrating cells relative to cells incubated in normoglycemic conditions. Bars and error bars refer to mean and SD of two experiments performed in duplicate.

Tumor microevironment plays a crucial role in cancer onset and progression. In particular, extracellular proteins of the tumor microenvironment or hypoxia may modulate the expression of mesenchymal markers [20]. To study the expression of ZEB2 and ZEB1 in shortage of nutrients conditions, we incubated Hey cells for 48 or 72 hrs in hypoglycemic conditions. We found that the treatment leads to a decreased ZEB2 and ZEB1 proteins expression, while the mRNAs levels appeared unaffected (Figure 5A and 5B). Interestingly, in the same experiments the expression of the mesenchymal factor vimentin showed a significant reduction (Figure 5C). The levels of the epithelial marker E-cadherin were almost undetectable in both normoglycemic and hypoglycemic conditions (data not shown).

Since HuR is a stress response protein [21–22, 17] and we found that it is a positive regulator of ZEB2 expression, we investigated if the observed reduction of ZEB2 expression in hypoglycemic conditions correlated with HuR binding and regulatory activity on ZEB2 mRNA. RIP assay revealed a reduction of HuR-ZEB2 mRNA complexes in hypoglycemic conditions (Figure 5D). In line with these results, the pulldown experiments indicated that in the same conditions HuR association decreased, both with the ZUTR probe and B2 probe (Figure 5E, see also scheme of Figure 3A). HuR expression was monitored in cytoplasmic extracts and a reduction of HuR protein levels was detected (Figure 5F). Taken together these findings confirmed a decrease of HuR-ZEB2 mRNA complexes in hypoglycemic conditions.

Next we investigated if HuR silencing further affected ZEB2 expression in hypoglycemic conditions, but no significant modulation was observed (Figure 5G).

Finally, the functional meaning of the described modulations was explored, analyzing the migration ability of Hey cells in hypoglycemic conditions. A marked decrease was observed (Figure 5H), allowing to hypothesize that HuR, ZEB2, ZEB1 and vimentin could be involved in the unpaired migration in hypoglycemic conditions.

Overall, the positive role of HuR in modulating ZEB2 expression was confirmed, as the decrease of HuR binding in hypoglycemic conditions correlated with reduced ZEB2 expression, with associated reduced migration ability.

### Nuclear ZEB2 is localized in tumor leading edge and co-localizes with cytoplasmic HuR

Analysis of ZEB2 and HuR expression was assessed by immunohistochemistry in a large series of ovarian cancer patients (Table 1). Evaluation of ZEB2 and HuR was firstly focused on the localization within the cells (nuclear versus cytoplasmic) and in the tumor layers (core versus edge).

**Table 1:** Clinico-pathological characteristics of our study population

Characteristics	Nr. of patients (%)
**All cases**	143
**Median Age (years, range)**	59 (35–83)
**Tumor histotype**	
Serous	104(72.7)
Endometrioid	20(14.0)
Other	19 (13.3)
**Grade**	
G2	28(19.5)
G3	98(68.5)
n.a.	23 (12.0)
**FIGO Stage at diagnosis**	
I–II	31(21.7)
III–IV	15 (78.3)
**Median CA125 at diagnosis (range, UI/ml)**	675 (11-9082)
**Ascites**	
Yes	85(59.4)
No	58 (40.6)
**Peritoneal carcinomatosis**	
Yes	85(59.4)
No	87 (60.8)
**Median Platinum-free interval**	
PFI ≤ 6 months	44(30.8)
PFI > 6 months	99 (69.2)

**Figure 6:**
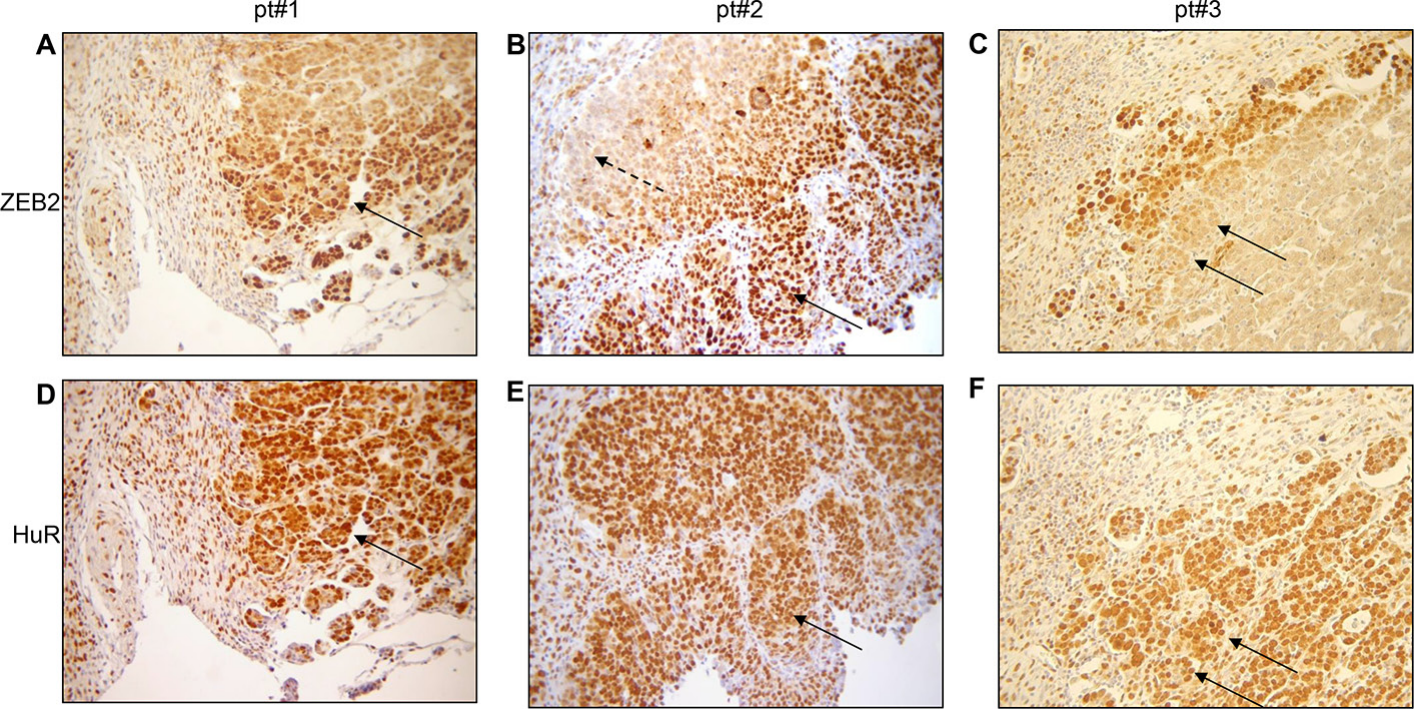
In tumor edge nuclear ZEB2 co-localizes with cytoplasmic HuR Representative immunohistochemical analysis in ovarian cancers for three patients (pt#1, pt#2, pt#3) showing nuclear ZEB2 staining in the edge (**A.** and **B.** continuous arrows), cytoplasmic ZEB2 staining in the inner core (B, dashed arrow) and nuclear and cytoplasmic HuR staining **D.** and **E.** in sequential slides from A and B. The weak ZEB2 staining in pt#3 **C.** is indicated by arrows, as the corresponding HuR staining **F.** in sequential slide.

The analysis of ZEB2 expression revealed that in the tumor edge the protein was mainly localized in the nucleus (Figure 6A, 6B, continuous arrows), whereas in the inner part of the tumor the staining, if present, was restricted to the cytoplasm (panel B, dashed arrow). The weak ZEB2 staining in a subset of tumors (e.g. pt#3, panel C) demonstrated the specificity of the obtained results. The morphology of the cells in the leading edge was not different from the morphology of the cells in the inner mass of the tumors, nevertheless the sharp nuclear localization of ZEB2 in the tumor front suggested a commitment for the EMT process. In the same subset of ovarian cancers, sequential immunohistochemical analysis for HuR protein expression and localization was performed. Interestingly, we observed that in cells where ZEB2 is localized in the nucleus HuR is localized both in the cytoplasm and in the nucleus, as observed in representative Figures 6D and 6E. These findings are consistent with the *in vitro* results indicating HuR as a positive regulator of ZEB2 expression.

### Concomitant high nuclear ZEB2 and cytoplasmic HuR expression correlates with poor prognosis

The expression of ZEB2 and HuR was analyzed in 143 women with high-grade serous (72.7%), advanced (78.3%) ovarian cancer, whose clinical features are summarized in Table 1.

**Figure 7:**
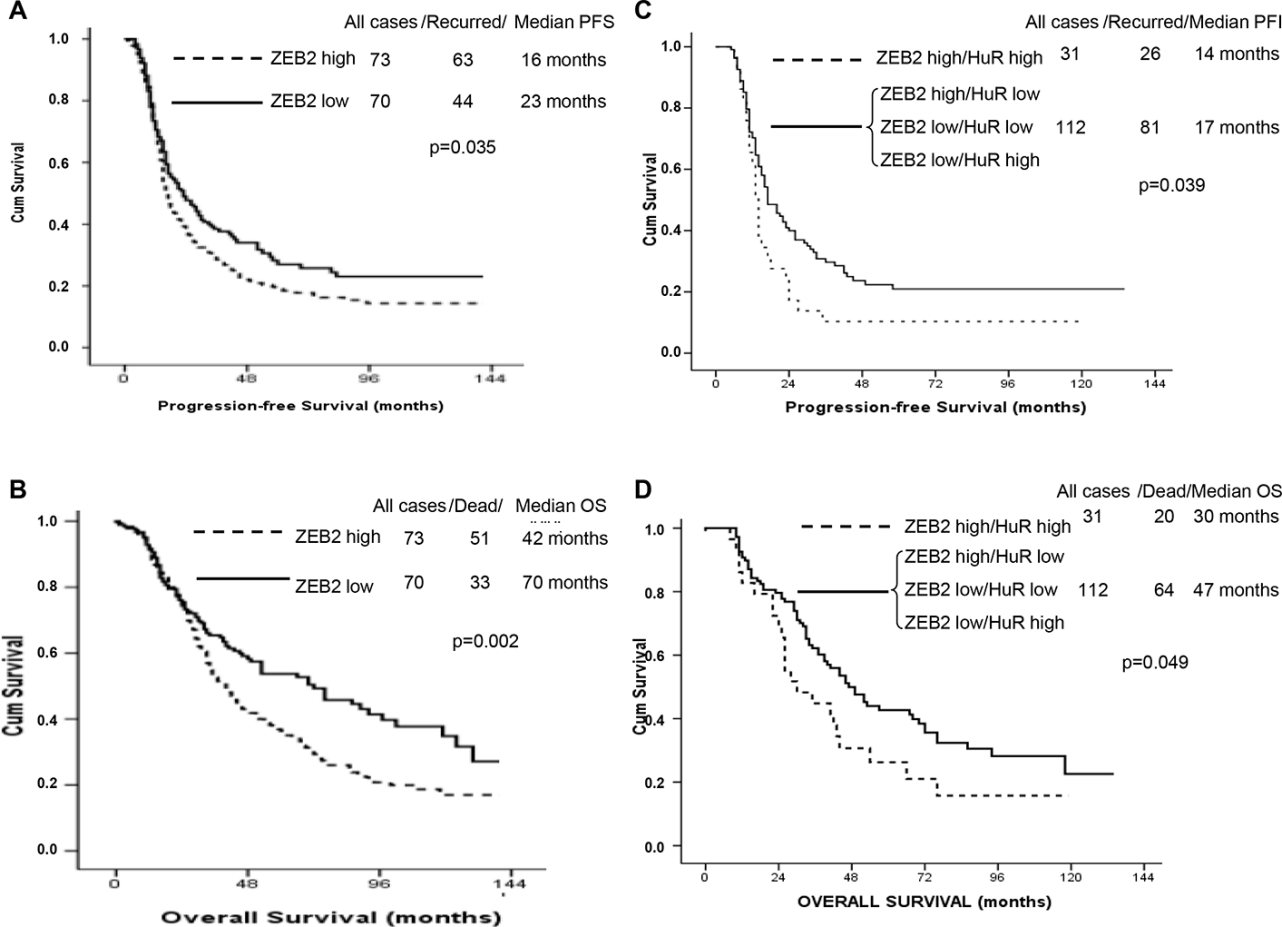
ZEB2 expression levels analysis in ovarian cancers and prognosis correlation Kaplan-Meier analysis of Progression-free Survival **A.** and Overall Survival **B.** for ovarian cancer patients according to ZEB2 mRNA expression. Kaplan-Meier analysis of Progression-free Survival **C.** and Overall Survival **D.** according to nuclear ZEB2 and cytoplasmic HuR protein expression, following categorization of patients in four groups, as indicated.

Firstly, analysis of ZEB2 mRNA was performed using the nanofluidic technology. As previously reported [23–24], the median served as the cut-off value to identify group of patients with high or low levels of ZEB2 mRNA. We observed a statistically significant decreased PFS (*p*-value = 0.035), and OS (*p*-value = 0.002) in patients with high expression levels of ZEB2 mRNA, compared with patients with low expression levels (Figure 7A, 7B).

Since ZEB2 expression is widely modulated at post-transcriptional level [7, this study] it was crucial to evaluate the expression levels of ZEB2 protein by immunohistochemical analysis (Figure 7C, 7D). Median expression nuclear and cytoplasmic levels of ZEB2 were 20 (0–90), and 0 (0–80) respectively. Cytoplasmic median IHC levels of HuR were 45, ranging from 0 to 100. Median levels were established as cut-off values to identify patients with high and low expression levels (see Materials and Methods). No correlation was observed between nuclear and cytoplasmic ZEB2, as well as cytoplasmic HuR expression levels and survival figures (PFS, OS; data not shown). As a further step, we divided the overall series in four groups, according with the combined expression levels of nuclear ZEB2 and cytoplasmic HuR. No correlation was observed between the nuclear ZEB2 and cytoplasmic HuR expression levels and the presence of peritoneal carcinomatosis. On the other hand, we demonstrated a statistically significant worse PFS (*p*-value = 0.039), and OS (*p*-value = 0.049) in women with high expression levels of both nuclear ZEB2 and cytoplasmic HuR, compared with the other groups. These findings indicated that in ovarian cancer the prognostic significance of ZEB2 protein relies on its nuclear expression and colocalization with cytoplasmic HuR.

## DISCUSSION

The plasticity of cancer cells underlies their capacity to adapt to the selective pressures they encounter during tumour development. Aberrant reactivation of EMT can promote cancer cell plasticity and foster both tumour initiation and metastatic spread. Such phenomenon is evident in ovarian cancer progression, where the shedding of cancerous cells from the ovary into the peritoneal cavity is the first step of the metastatic spread [3]. In this context, our study of the mesenchymal factor ZEB2 expression, function and regulation aimed to provide new elements for a deeper knowledge of cancer dissemination and a more effective therapeutic approach.

In a panel of ovarian adenocarcinoma cell lines we found that the different epithelial and mesenchymal markers display a wide degree of expression variability. These findings likely reflect the progressive nature of the EMT process and represents the relevant heterogeneity of phenotypes observed *in vivo*. The Hey cell line express ZEB2 protein and lowest levels of miR-200c and miR-141, suggesting that these two members of the miR-200 family could have a critical role in ZEB2 repression in other ovarian cancer cells. Notably, these miRNAs are clustered on chromosome 12p12.31 and transcribed from the same promoter, thus suggesting that they can be co-regulated at transcriptional level.

It is widely accepted that ZEB2 is involved in cancer invasion in different tumors, as glioma and renal cell carcinoma [11–12, 7]. Our findings demonstrated that ZEB2 knock-down in Hey ovarian adenocarcinoma cells impaired migration, invasion and anchorage-independent cell growth, indicating a pivotal role for ZEB2 in progression of ovarian carcinogenesis. We noted that Hey cells do not restore E-cadherin expression in response to decreased ZEB2 or levels, and the expression of miR-200s were also unchanged (unpublished data). Similar results were obtained with ZEB1 knockdown (unpublished data), in line with Cochrane at al. reporting that in Hey cells knocked-down for ZEB1 no expression of E-cadherin was observed [25]. Therefore, it is possible to hypothesize that in Hey cells different factors contribute to E-cadherin repression and that either ZEB1 or ZEB2 silencing is not sufficient to progress toward a full epithelial phenotype.

The study of ZEB2 expression regulation is of pivotal importance to understand the cancer progression. In the present study we demonstrated that ZEB2 mRNA expression is modulated by the RBP HuR, which induces ZEB2 protein expression and affects cellular migration. Our results suggested that HuR could affect cellular migration through modulation of ZEB2 and ZEB1 genes expression, underlining its role in carcinogenesis progression.

Several stress signals, including oxidative stress, amino acid starvation, and polyamine depletion result in the cytoplasmic accumulation of HuR and the subsequent stabilization of target mRNAs [17–18]. Other stress signals, such as heat shock, hypoxia, and staurosporine, were reported to reduce HuR expression through proteasomal degradation or caspase-mediated cleavage mRNAs [26–27]. Our findings indicated that in hypoglycemic conditions the migration ability of Hey cells is reduced, along with decreased expression of cytoplasmic HuR, nuclear ZEB2 and ZEB1, and vimentin. The reduced HuR binding to the 3′UTR of ZEB2 mRNA may depend on the decreased cytoplasmic HuR expression, but also on the interaction with different factors, such as miRNAs or other RNA-binding proteins, which affect the binding and the regulatory activity of HuR on ZEB2 mRNA.

ZEB2 gene expression has been shown to be modulated at post-transcriptional level by the DNA/RNA binding YB-1 in breast epithelial cells [28], and by an increasing number of miRNAs, in addition to the miR-200 family [29–33]. Compelling evidences demonstrated that miRNAs and RNA-binding proteins functionally cooperate in modulation of shared targets [18–19, 24]. Binding of RBPs near miRNA target sites can potentially regulate miRNA function either directly by affecting miRNA binding or indirectly through a switch in RNA secondary structure family [19]. Intriguingly, the HuR binding region on ZEB2 mRNA 3′UTR includes seven binding sites for the miR-200 family members (data not shown), therefore it can be speculated that HuR and miRNAs may interplay on ZEB2 3′UTR regulating ZEB2 expression, thus contributing to modulate EMT progression and invasivity of cancer cells.

The discovery that HuR silencing decreases ZEB1 protein expression in our system suggests that HuR is involved in modulation of this gene. This finding is in line with the emerging concept of “mRNA regulons”, which hypothesizes that groups of mRNAs encoding functionally related proteins act regulating their own expression at post-transcriptional level, through a ribonucleoprotein-driven mechanism [34–35]. Previous reports showed that HuR enhances the expression of different proteins promoting invasion and EMT, including SNAIL, MMP-9, uPA and the uPA receptor [18]; thus HuR appears to be a key factor in cancer progression.

Our immunohistochemical findings provide additional evidences on ZEB2 role in tumor progression. To our knowledge, this is the first study demonstrating a clear nuclear localization of ZEB2 protein in cancer invasive front. This nuclear localization strongly suggests that as a transcription factor ZEB2 exploited its regulatory activity on target genes specifically in the leading edge of the tumor, providing a commitment for the EMT process and an increased invasion ability. In colorectal cancer a graduated, often concentric epithelial differentiation is clearly detectable [36], and ZEB2 was found overexpressed of at the invasion front, predominantly in the cytoplasm [37]. Similarly, in ovarian carcinoma the presence of tumor layers with different ZEB2 expression could reflect the phenotypic plasticity within the same tumor and support the pivotal role of EMT-TFs during ovarian carcinogenesis. The localization of cytoplasmic HuR in the tumor edge confirmed the pivotal role of this RNA-binding protein in tumor progression.

We described that ZEB2 was expressed in the cytoplasm in the inner part of the ovarian tumors. This cellular localization was observed in multiple tumor tissue arrays, colorectal cancer, glioma, renal cell carcinoma [37–38, 11–12], suggesting that cytosolic ZEB2 may play additional functions. Interestingly, ZEB1 was observed in the cytosol in endothelial cells, bound to CalDAG-GEFIII, an R-Ras activator [39]. Alternatively, cytosolic localization of ZEB2 may indicate a fast turn-over of the protein, with a transient nuclear expression reflecting the highly dynamic plasticity of the EMT progression.

The prognostic meaning of ZEB2 expression in ovarian cancer patients is a significant finding of the current study. We showed that ZEB2 mRNA overexpression is associated with poor outcome, and that concomitant overexpression of nuclear ZEB2 and cytoplasmic Hur at protein level correlates with shorter progression-free-survival and overall survival in ovarian cancer patients.

A similar correlation between ZEB2 expression and prognosis was found in different cancer types [11–12, 37–38, 40–41], including ovarian cancer [9, 42–43]. Our analysis was performed on a large subset of patients and demonstrated the importance of the evaluation not only of ZEB2 mRNA levels, but also of ZEB2 protein levels, subcellular localization and colocalization with the regulatory factor HuR. In addition, since tumor samples could include neoplastic cells with different EMT profiles, our study highlights the advisability to study mRNAs and proteins expression in different tumor layers.

In conclusion, our findings provide evidence for a role of ZEB2 and HuR in EMT progression and development of an aggressive phenotype in ovarian cancer. Considering that EMT is controlled by a network of transcriptional, post-transcriptional and post-translational regulators, the definition of the gene regulatory network will be fundamental to understanding cancer progression. A better comprehension of the mechanisms underlying the cancer cell plasticity conferred by EMT programmes might facilitate the design of therapeutic interventions aiming to target selected signaling pathways and prevent cancer initiation and progression.

## MATERIALs AND METHODS

### Cell cultures and reagents

A2780, SKOV3 and OVCAR-3 cells were purchased from the European Collection of Cell Cultures. Hey cells were donated by Susan Horwitz (Albert Einstein Medical College). SKOV6 and OV2774 were kindly donated by Dr. Kunle Odunsi (Roswell Park Cancer Institute, Buffalo NY). Culture media were selected according to the suggestions of European Collection of Cell Cultures. Glucose-free RPMI (Gibco) medium was used for hypoglycemia experiments. Growth experiments were performed as previously described [Raspaglio 2010]. Silencing of ZEB2 gene expression was obtained by transfection with Transfectin (Bio-Rad) and specific siRNAs (siZEB2), while a non targeting siRNA pool was used as control (siC) (Dharmacon, Lafayette, CO). Transfection with siHuR and siC oligonucleotide duplex were performed as previously described [22].

### Real-time quantitative PCR and western blotting

Quantitative PCR on mRNAs was performed as previously described [22]. MiRNAs reverse transcription and PCR reactions were performed on Trizol (Invitrogen, Carlsbad, CA, USA) isolated total RNAs using TaqMan MicroRNA Assays kit (Applied Biosystems, Foster City, CA, USA) as described [24].

Western blots were done on total lysates or on nuclear/cytoplasmic fractions. Total cellular proteins were obtained lysing the cells with EB buffer [20 mmol/L Tris-HCl (pH 7.4), 5 mmol/L EDTA, 150 mmol/L NaCl, 10% glycerol, 1% Triton X-100] in the presence of proteases and phosphatase inhibitors. Nuclear proteins were obtained incubating the cells with A buffer [10 mmol/L Hepes (pH 7.4), 10 mmol/L KCl, 0, 1 mmol/L EDTA, 0, 1 mmol/L EGTA] 15 minutes in ice, then adding 0, 66% Nonidet NP-40 and incubating for additional 2 minutes. After centrifugation the cytoplasmic extract was recovered and the nuclear pellet was lysed with B buffer [20 mmol/L Hepes (pH 7.4), 0, 4 mol/L NaCl, 1 mmol/L EDTA, 1 mmol/L EGTA, 1% Triton X-100] 30 minutes in ice. For the Western blot analysis the following antibodies were utilized: anti-ZEB2/SIP1 (1:1000, Abcam), anti-ZEB1 (1:500 Santa Cruz, Santa Cruz, CA), anti-E-Cadherin (1:1000, Cell Signaling), anti-vimentin (1:500, Santa Cruz, Santa Cruz, CA), anti-HuR (1:500, Santa Cruz, Santa Cruz, CA), anti-β-actin (1:5000, Sigma, Saint Louis, MO). Blots were visualized by enhanced chemiluminescence procedures (Amersham, GE-Healthcare, Buckinghamshire, UK) as described by the manufacturers.

### Wound healing assay

Hey cells were transfected with siZEB2 or siC oligos (see above) and incubated for 48 hours. Then wounds were created in 80% confluent cells in 100 mm-plate plates using a 200-μl pipette tip and the cells were rinsed with medium to remove any free-floating cells and debris. Culture were incubated at 37°C and closure of the wound healing was monitored and photographed in five different fields in each plate.

### Transwell migration assays

Transwell migration assays were performed on Hey cells transfected with siZEB2 oligos (see above) for 48 hours and serum starved for 16 hours. Then 1000 cells were plated in 0.5 mL media without serum in the upper chamber of BD BioCoat Control Insert Chambers (24-weel plate with 8 μm pore size). In the lower chamber 0.5 mL media containing 10% FBS was used as an attractant. After the cells were incubated for 5 hours at 37° in a 5% CO_2_ atmosphere, the inserts were washed with PBS, and cells on the top surface of the insert were removed with a cotton swab. Cells adhering to the lower surface were fixed with ice-cold methanol for 10 minutes, stained with 1% Crystal violet for 20 minutes, extensively washed with ddH_2_O and counted under a microscope.

### Anchorage-independent growth assay

Hey cells were transfected with siZEB2 or siC oligos (see above) and incubated for 48 hours. Cells were then mixed in 2 × RPMI 1640 with an equal volume of soft agar (Sigma) to give a final solution of 0.3% agar, 1 × RPMI 1640, and 10% FBS, and the cell–agar mixture was added to the top of the cell-free bottom layer with 0.6% agar. 8000 cells/well were plated in six well plates. After six days, viable colonies larger than 0.1 mm were counted.

### Biotinylated RNA probe, pull-down assay and ribonucleoprotein immunoprecipitation

Different sequences of the 3′UTR of ZEB2 mRNA (NT_014795) were amplified and cloned in pGEM-T Easy Vector(Promega). The following primers were used: for the probe Z-UTR forward 5′-CCTCTAGAGAAGAC AATATGGAAGATGGCATG-3′ and reverse 5′-CCTCTA GAGCATAAAGCATGTTA CATGTTAATGG-3′; for the probe A forward 5′-CCTCTAGAGAAGACAATATG GAAGATGGCATG-3′ and reverse 5′- TGCATTGTAG TGCGAGCACATT-3′; for the probe B forward 5′- TCAGTATTATGATTCCTCTG-3′ and reverse 5′-AT ACTGTACACTACAGTATG-3′; for the probe C forward 5′- TATAGTTCTTCAATATATAGAT-3′ and reverse 5′- CCTCTAGAGCATAAAGCATGTTACATGTTAATGG -3′; for the probe D forward 5′-GCCATCCTTGTA CAGTGTTAAG-3′ and reverse 5′-GTCGAAAATACA GTGTTTTCAC-3′; for the probe B1 forward 5′- GCCATCCTTGTACAGTGTTAAG-3′ and reverse 5′- ATACTGTACACTACAGTATG-3′; for the probe B2 forward 5′-TATTACACCAAACTGTTTTTGC-3′ and reverse 5′-ATACTGTACACTACAGTATG-3′; for the probe B3 forward 5′-CTGTGAAGGAACTTGAAGTG-3′ and reverse 5′-GCATATAAGGCTTTAAAACCA-3′ The plasmids were linearized and *in vitro* transcribed using T7 RNA polymerase (Invitrogen), in the presence of ^14^C-biotynilated CTP (Invitrogen). 269-nt long negative control probe was obtained by the empty pGEM -3Zf (+) vector.

The pull-down assays were performed on cytoplasmic extracts obtained as described above, with A buffer supplemented by mmol/l DTT. Potassium acetate was added to a final concentration of 90 mmol/L and cytoplasmic extracts were precleared with streptavidin-conjugated agarose (Upstate) in binding buffer [10 mmol/L HEPES (pH 7.5), 90 mmol/L potassium acetate, 1.5 mmol/L MgCl_2_, 2.5 mmol/L dithiothreitol, 0.05% NP40, protease and phosphatase inhibitor cocktail], in the presence of RNase inhibitor (Roche; 100 units/mL) and yeast tRNA (20 μg/mL; Ambion), for 1 hour at 4°C with rotation. After centrifugation at 8,000 × *g* for 1 minute, the supernatants were mixed with *in vitro* transcribed biotinylated probes or negative control probe, and the mixtures were incubated for 1 hour at 4°C. Protein and biotinylated RNA complexes were recovered by addition of streptavidin-conjugated agarose at 4°C for 2 hours, with rotation. The precipitated complexes were extensively washed with binding buffer, boiled in SDS-PAGE sample buffer, and resolved by gel electrophoresis followed by Western blotting with anti-HuR antibody.

Ribonucleoprotein immunoprecipitation with anti-HuR antibody (Santa Cruz) or nonspecific IgG (Bio-Rad) were performed as previously described [22] and analyzed by real-time PCR.

### Immunohistochemistry

The expression of HuR and ZEB2 was immunohistochemically assessed in a series of 143 ovarian cancers admitted in the Gynaecologic Oncology unit of our department.

Immunostaining was performed on 3 mm paraffin tissue section mounted on poly-l-lysine-coated slides and dries at 37°C overnight. After the slides were deparaffinized in xilene and rehydrated conventionally, the endogenous peroxidase was blocked with 3% H_2_O_2_ in H_2_O for 5 minutes. Antigen retrieval procedure was performed by microwave oven heating in 10mM citric acid, pH 6.0 (2 times for 4 minutes) for HuR and by microwave oven heating in EDTA pH8 for ZEB2. To reduce non specific binding the sections were incubated with 20% normal goat serum for 30 minutes at room temperature. Cells expressing HuR and ZEB2 were identified after ON incubation at 4°C by using the monoclonal antihuman HuR antibody (3A2; 1:1000; Santa Cruz Biotechnology) and polyclonal antihuman ZEB2 antibody (1:150; Abcam) respectively. Detection was evaluted by a labelled polymer EnVision-mouse+ System-HRP (DAKO, Carpinteria, CA, USA) for HuR and by a labelled polymer EnVision-rabbit+ System-HRP (DAKO, Carpinteria, CA, USA) for ZEB2, for both 30 minutes at room temperature. Diaminobenzidine was used as a chromogen (DAB substrate System, DAKO). Sections were counterstained with haematoxylin, dehydrated, cleared in xylene and mounted with Eukitt. Negative control was obtained by omission of the primary antibody. Positive control for HuR and ZEB2 was represented by sections taken from the colon cancer and breast cancer respectively.

The analysis of all tissue sections was done without any prior knowledge of clinical parameters by GFZ and EM by means of light microscopy. The proportion of immunostained tumour cells was scored at low magnification (5X objective lens) by evaluating the entire tumour area.

### Nanofluidic analysis of mRNA expression

FFPE samples were obtained from ovarian cancer that had been preserved between 2000 and 2008 following the approved Danbury Hospital Internal Review Board protocol. FFPE samples were cut to 10 μm thickness and two tissue slices were put into a 1.5 ml tube. One milliliter of xylene was added for deparaffinization followed by mixing twice with a high speed vortex for 3 min at room temperature. Total RNA was then automatically extracted with the QIAcube using the Qiagen miRNeasy FFPE kit (Valencia, CA) following manufacturers' protocols. The RNA from the cell line A2780 was automatically extracted with the QIAcube using the Qiagen miRNeasy kit (Valencia, CA) following manufacturer's protocols. RNA quantity and the quality were assessed by Agilent 2100 Bioanalyzer (Agilent Technologies, Santa Clara, CA). Analysis was carried out using the 48.48 dynamic array (Fluidigm Corporation, CA, USA) and a Biomark platform following the manufacturer's protocol.

### Statistical analysis

Overall survival (OS) and progression free survival (PFS) were calculated from the date of diagnosis to the date of progression/death or date last seen. Medians and life tables were computed using the product-limit estimate by the Kaplan-Meier method and the Wilcoxon test was employed only to assess statistical significance. Multivariate analysis assessed the clinical role of ZEB2 and HuR pattern of staining in a model including additional significant variables in univariate analysis such as (age, stage and histotype) using the Cox proportional hazards model and nonparametric testing with the Kruskal Wallis test. Statistical analysis was carried out using JMP9 (SAS).

## SUPPLEMENTARY FIGURE


