# Supplementary material for: Role and prognostic significance of the epithelial-mesenchymal transition factor ZEB2 in ovarian cancer
Source: Oncotarget. 2015 May 15;6(22):18966–79. doi: 10.18632/oncotarget.3943 (PMC4662468; doi:10.18632/oncotarget.3943)
Supplement: Supplementary file 1 [file oncotarget-06-18966-s001.pdf]

SUPPLEMENTARY FIGURE

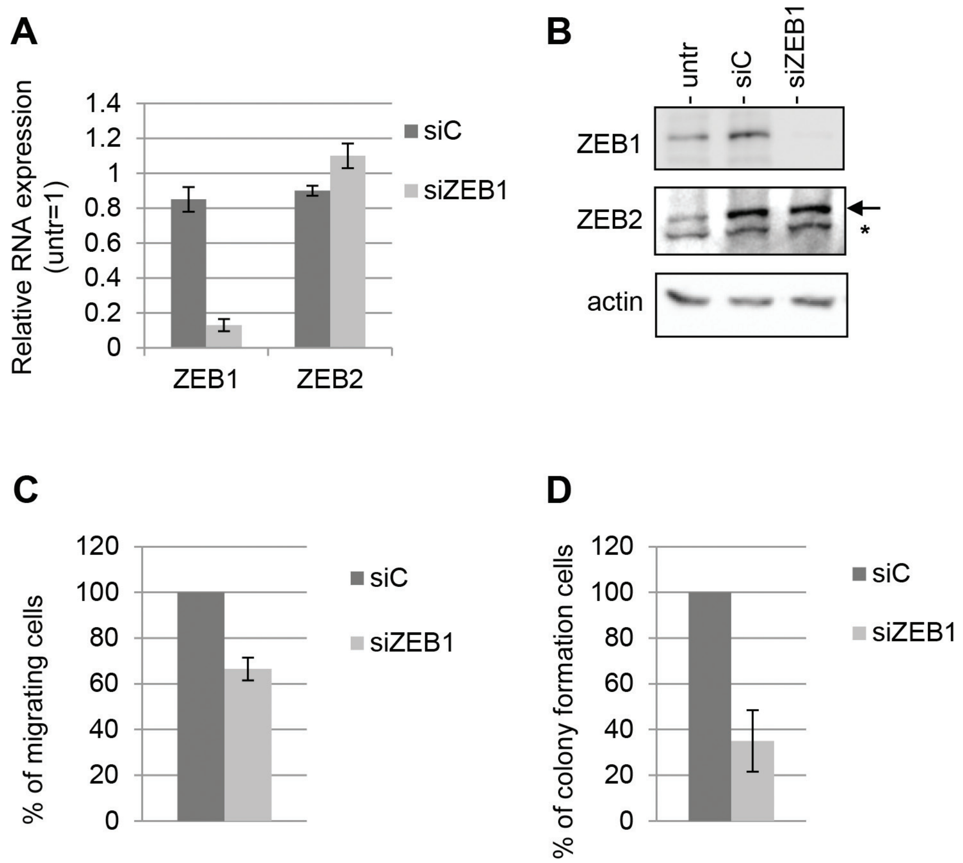

Supplementary Figure 1: ZEB1 knockdown impairs migration and anchorage-independent cell growth in Hey cells. The experiments were performed on Hey cells untrasfected, trasfected with siZEB2 oligos or with siC oligos and incubated for 48 hours. **A.** Q-PCR analysis of ZEB1 and ZEB2 mRNAs expression, values are expressed relative to the levels measured in untrasfected cells (=1). Bars and error bars refer to mean and SD of two experiments performed in triplicate. **B.** Representative Western Blots analysis of ZEB2 and ZEB1 protein expression on nuclear extracts. The arrow indicated ZEB2 protein band and the asterisk indicated an unspecific band. Actin probing served as loading control. **C.** Transwell migration assays. The values are expressed as percentage of migrating cells relative to siC-transfected cells. Bars and error bars refer to mean and SD of two experiments performed in duplicate. **D.** Anchorage-independent cell growth assays. The values are expressed as percentage of colonies relative to siC-transfected cells. Bars and error bars refer to mean and SD of two experiments performed in duplicate.
